# Congenital Heart Diseases: Genetic Risk Variants and Their Methylation Status

**DOI:** 10.3390/genes13112115

**Published:** 2022-11-15

**Authors:** Melissa Calzada-Dávila, Geovana Calvo-Anguiano, Laura E. Martínez-de-Villarreal, José J. Lugo-Trampe, Sandra M. González-Peña, Patricia R. Ancer-Rodríguez, María D. Hernández-Almaguer, Luis D. Campos-Acevedo

**Affiliations:** 1 Genetics Department, Hospital Universitario “Dr. José Eleuterio González” and Medicine Faculty, Autonomous University of Nuevo León, Monterrey 64460, Mexico; 2 Clinical Nutrition, Hospital Universitario “Dr. José Eleuterio González” and Medicine Faculty, Autonomous University of Nuevo León, Monterrey 64460, Mexico; 3 Medicine Faculty, Autonomous University of Baja California, Mexicali 21000, Mexico

**Keywords:** folic acid intake, ventricular septal defects, atrial septal defects, congenital heart disease, methylation status, epigenetics, *AXIN1*, *MTHFR*, *TBX1*, *TBX20*

## Abstract

(1) Background: The interaction between single nucleotide variants (SNVs) associated with congenital heart diseases (CHDs) and their gene methylation status has not been well researched. The aim of the present study was to determine if there is a relationship between the methy lation status (MS) of genes and the allelic variants associated with CHDs. (2) Methods: Seven SNVs of the genes *AXIN1*, *TBX1*, *TBX20*, and *MTHFR* were selected from the literature. DNA extraction, genotyping, and a methylation analysis were performed on healthy subjects and subjects with CHDs. (3) Results: Twenty-two subjects with CHDs were selected as the case group (15 with ventricular septal defects (VSDs) and 7 with atrial septal defects (ASDs)), and 44 healthy subjects comprised the control group. The *MTHFR* and *AXIN1* genes were hypermethylated in the control group when compared to the case group. When analyzed separately, those with atrial septum defects exhibited greater methylation, except for the gene MTHFR where there were no differences. Only the alternate alleles of MTHFR showed a significantly different methylation status in those without cardiopathy. (4) Conclusions: The *MTHFR* and *AXIN* genes were hypermethylated in the control group; however, only the alternate alleles of *MTHFR* (rs1801133 and rs1801131) showed a significantly different methylation status.

## 1. Introduction

The global prevalence of congenital birth defects is about 2–3% [1]. Congenital heart disease (CHD) encompasses a group of anomalies of the heart and the great vessels. These defects are the most common type of human malformations with a worldwide frequency of 7 in every 1000 live newborns [2]. Ventricular septal defects (VSDs) account for 20% of all CHDs and are the third most common isolated defect, after patent ductus arteriosus (PDA) and atrial septal defects (ASDs) [3].

In Mexico, the frequency of CHD is unknown; however, it is estimated that each year, between 12,000 and 16,000 children are born with CHD, and it is the second most prevalent cause of death in Mexican children under five years old [4].

As with most human malformations, CHD is a complex, multi-factorial disease that arises through the interaction of genetic and environmental factors [5], with a concordance and a heritability of 40–60% and 37%, respectively [6,7]. The early diagnosis and prevention of congenital birth defects have improved worldwide, and the survival rate has increased, such that the study of genetic and environmental contributing factors has gained interest among public health researchers [8].

A recent series of studies by Homsy et al. (2015) and Jin et al. (2017) estimated that only 2% of patients with isolated CHD had pathogenic mutations identified by whole-exome sequencing. Interestingly, some genes associated with CHD are involved in morphogenesis, chromatin modification, and transcriptional regulation; however, for the majority of cases, the causes remain unknown [9,10,11]. Multiple genes and gene interactions may be involved in nonsyndromic and isolated CHDs. The development of new techniques, such as genome-wide sequencing (GWS) and exome sequencing, has enabled researchers to find gene variants that may confer a risk of CHD in specific populations.

In their meta-analysis, Yin et al. (2011) described the relationship between *MTHFR* and CHD, reporting a significant association in various populations (Europeans, Americans, Asians, and Hispanics) [12]. A previous study by our group (Dolores et al. (2019)) in a northeastern Mexican population analyzed the association of 14 SNVs of 8 genes (*TBX1*, *TBX20*, *ASTX-18-AS1*, *AXIN1*, *MTHFR*, *NKX2.5*, *BMP4*, and *NFATc1.*) and found a significant association with *AXIN1* and *TBX20* but not with *MTHFR* [13]. There are currently few reports on methylation status however, to advance the diagnosis, understanding, and treatment of CHD, the extra-genomic factors critical for heart development need to be uncovered [9].

Epigenetics is an extra-genomic mechanism that is capable of regulating gene expression through the control of transcription and translation without changing the underlying nucleotide sequence. Epigenetic modifications include DNA methylation, histone modification, and non-coding RNAs and can be inherited by daughter cells during cell division. An increasing body of clinical and experimental research has identified aberrant epigenetic patterns in several polygenic diseases, including CHD [14].

There are few reports regarding gene methylation and risk for congenital defects; therefore, the aim of the present study was to determine if the methylation status (MS) of the SNVs reported to be associated with CHD is related to the risk for developing a CHD. The SNVs studied were rs370681, rs1805105, and rs12921862 in *AXIN1*; rs4720169 in *TBX20*; rs41260844 in *TBX1*; and rs1801133 and rs1801131 in *MTHFR*.

## 2. Materials and Methods

### 2.1. Study Population

Cases were recruited from two local hospitals: Hospital Universitario “Dr. José Eleuterio González” and Hospital Regional Materno-Infantil de Alta Especialidad, in Nuevo León, Mexico. The study was approved by the Ethics and Research Committee of the Facultad de Medicina, Universidad Autónoma de Nuevo León (protocol code GN19-00001), according to the code of ethics of the World Medical Association (Declaration of Helsinki). Informed consent was obtained from all subjects involved in the study, as established in the regulations of the general health law of Mexico.

This study included all children with a diagnosis of VSD and ASD. Patients with a positive family history of CHD were excluded. Patients with other malformations that suggested a chromosomal or monogenic etiology for CHD and subjects with additional symptoms (genetic syndromes associated with CHD) were excluded. The controls were DNA samples previously collected from healthy children (*n* = 44) that were stored in the DNA bank of the Department of Genetics of the Hospital Universitario “Dr. José Eleuterio González” (Table 1).

### 2.2. Single Nucleotide Variant (SNV) Selection

A previous study carried out by our group (Hernández-Almaguer et al. (2019) [13]) found statistically significant differences in the SNVs of the *TBX20* (rs4720169) and *AXIN1* (rs370681, rs1805105, and rs12921862) genes between cases and controls with a significant increase in the risk of CHDs in the population from northeast Mexico (Hernández-Almaguer (2019) [13]). The *MTHFR* (rs1801133 and rs1801131) and *TBX1* (rs41260844) genes were selected from the existing literature [14].

### 2.3. DNA Extraction and Genotyping

DNA was extracted from blood samples using a commercial Wizard Genomic DNA purification kit (Promega, Madison, WI, USA), following the manufacturer’s procedures. DNA quality and quantity were also verified by spectrophotometry (UV–Vis) using a NanoDrop™ 8000 (Thermo Fisher, Wilmington, DE, USA). A set of previously validated SNVs was used for rhAmp in a Step One Plus (Thermo Fisher) with a reaction volume of 10 μL. SNV allelic discrimination was performed using FAM and YAKIMA.

### 2.4. Quantitative Methylation Analysis

A methylation analysis of specific gene promoters was performed using EpiJET™ DNA Methylation Analysis Kits (MspI/HpaII) (Thermo Scientific™, Vilnius, Lithuania), following the manufacturer’s instructions. A DNA sample was divided into three tubes, and each was digested with a different endonuclease cut from either: (1) methylated DNA; (2) unmethylated DNA; or (3) undigested DNA. Methylated and unmethylated DNA controls were also included. We incubated the samples for 1 h at 37 °C.

Quantitative PCR (qPCR) was used to estimate the methylation level of the *MTHFR*, *TBX1*, *TBX20*, and *AXIN1* gene promoters, using Sybr Green and the primers specified in Table 2. The reactions were carried out in a final volume of 20 μL, containing 40 ng of digested DNA. qPCR was performed in a Step One Plus thermocycler (Applied Biosystems™, Foster City, CA, USA). The thermocycler protocol was as follows: DNA denaturation by incubation at 95 °C for 10 min followed by 40 cycles of 95 °C for 15 s, 60 °C for 30 s, 72 °C for 30 s, and a final extension at 72 °C for 2 min. The methylation status of the promoters of the genes of interest was analyzed using a Step One Real-Time PCR System (Applied Biosystems™, Foster City, CA, USA). Methylation was quantitatively expressed as the percentage of methylated cytosines over the total number of methylated and unmethylated cytosines.

### 2.5. Data Analysis/Statistical Analysis

Genotype and allele frequencies were obtained. For each SNV, deviation from the Hardy–Weinberg equilibrium (HWE) was estimated using the HWE program (https://gene-calc.pl/hardy-weinberg-page, accessed on 22 October 2020).

Data normality tests were performed. To determine the statistical differences between the cases and controls in terms of qualitative variables, a chi-square test was performed, while the Mann–Whitney U test was used for the quantitative variables. OR values were obtained to determine the risk variants in our population. The statistical analysis was performed using the SPSS v.22 statistical package (SPSS Inc.; Chicago, IL, USA). Probability values less than 0.05 (*p* < 0.05) were considered statistically significant.

## 3. Results

Fifty children with CHD were recruited; from these, 15 subjects with VSD and 7 subjects with ASD were selected for the study (*n* = 22; 44% of all CHDs).

### 3.1. Allelic and Genotype Frequencies and Association Analysis

The risk variants for CHD found in our population included rs12921862 of *AXIN1* (*p* = 0.00) and rs4720169 of *TBX20* (*p* = 0.005), although the latter was not in the Hardy–Weinberg equilibrium (HWE). The remaining variants did not show an increased risk. The variant rs12921862 of *AXIN1* had an OR of 69 (CI: 12.87–311.64, *p* = 0.000) for allele A (instead of the reference allele C).

### 3.2. Methylation Status: Cases vs. Controls 

In our analysis of methylation status, significant differences were observed between healthy children and children with VSDs and ASDs in terms of *MTHFR* and *AXIN1* (9.44 ± 37.9 vs. 4.48 ± 4.63, *p* = 0.041 and 96.5 ± 39.8 vs. 73.81 ± 35.6, *p* = 0.036, respectively), with relative hypermethylation in the control group (Table 3).

Methylation status was also compared between cases with VSDs vs. cases with ASDs. Significant differences were found in *AXIN1* (*p* = 0.001), *TBX20* (*p* = 0.017), and *TBX1* (0.001), with relative hypomethylation in the VSD group (Table 4).

### 3.3. Methylation Status: Wild-Type vs. Risk Variant

The case group (*n* = 22) and the control group (*n* = 44) were subdivided into those that carried the alternate alleles and those with the wild-type allele for each of the seven SNVs, and their MS was compared. Significant differences were found in rs1801131 and rs1801133, with a relative hypermethylation in healthy children who carried the alternate allele (*p* = 0.004 and *p* = 0.007, respectively) (Table 5).

## 4. Discussion

Similar to the previous study by Dolores-Almaguer (2019) [10], it was found that the SNVs associated with high risk in our population were rs12921862, with an OR for allele A of 63 (*AXIN1*), and rs4720169, with an OR for allele A of 7.6 (*TBX20*); however, in our study, *TBX20* in the control population was not in the HWE.

Despite these high ORs, the presence of these risk SNVs is neither sufficient nor necessary to produce congenital heart disease; thus, we sought to discover differences in MS associated with genotype that could be contributing to either the production or prevention of CHDs.

When comparing the MS between cases and controls, we found a statistically significant hypermethylation of the *AXIN1* (*p* = 0.036) and *MTHFR* (*p* = 0.041) genes in the control group, which could suggest that hypermethylation is a protective mechanism against CHD. Relative hypermethylation was also observed for the rest of the genes in the control group; however, the differences observed were not statistically significant.

When comparing the MS between the four subgroups, a genotype-dependent difference in methylation was observed only for *MTHFR*. MTHFR catalyzes the synthesis of 5-methyltetrahydrofalate, the methyl donor for the synthesis of methionine from homocysteine and the precursor of S-adenosyl-l-methionine, and DNA methylation is catalyzed by methyltransferases that use the universal methyl donor S-adenosyl-l-methionine [15]. We also observed the hypermethylation of rs1801131 in those controls with the alternate allele, which supports the notion that hypermethylation is a protective factor in the generation of congenital heart disease.

Previous studies have described differences in methylation patterns between healthy subjects and subjects with CHD. Studies comparing DNA methylation status in newborns with aortic valve stenosis, tetralogy of Fallot, and VSDs have identified several aberrant patterns [16,17,18]. However, the interaction between the gene variants associated with CHD and their MS has been less well researched.

The protective effect of periconceptional folate in preventing neural tube defects is widely recognized [19]. In a study by González-Peña (2021), the interaction between nutrients and epigenetics involved in congenital heart disease was explored. Folic acid intake was found to be significantly higher during the first trimester in mothers of healthy children vs. that of mothers of children with congenital heart disease [20]. We propose that this ingestion of folic acid could provide the necessary methyl groups that are needed for adequate gene expression, allowing for the correct modulation of the genes for heart development, and, thus, could be a protective factor for CHDs, as some researchers have already begun to report [21,22,23,24,25].

As a limitation, methylation can be both a contributing factor and a consequence of disease status. It would be interesting to investigate potential causal pathways using risk variant genotypes as instruments.

## 5. Conclusions

The methylation status of genes plays a role in the development of congenital heart defects. In our population, the methylation status of the *MTHFR* gene risk variant was a protective factor for congenital heart disease, as it was significantly higher in controls without CHD.

The risk association for CHD and the rs12921862 and rs4720169 in *AXIN1* and *TBX20*, respectively, was independent of the MS of their promoter.

## Figures and Tables

**Table 1:** Demographic characteristics of the cohort by case and control.

	Cases		Controls		*p*
	Male	Female	Male	Female	
Sex	12	10	20	24	
Type of CHD					
ASD	5	2	0	0	
VSD	10	5	0	0	
Weeks of gestation					
<37	4	4	1	4	0.0122
38–41	10	4	19	20	
>42	0	0	0	0	
Weight					
<2500 g	2	3	0	0	0.023
2500–3500 g	10	3	14	21	
>3500 g	2	2	6	3	
Height					
<48 cm	6	6	2	1	0.0001
48–50 cm	4	0	8	14	
>50 cm	4	2	10	9	
Maternal folic acid intake					
Preconceptional	0	3	0	0	0.0071
During pregnancy	10	7	20	24	
None	2	0	0	0	
Origin	Northeastern Mexico				

For the variables weeks of gestation, weight, and height, all with a normal distribution, *p* values were obtained using an unpaired student’s *t* test. For folic acid intake, the *p* value was obtained using chi-square analysis. P.05 statistical analysis was performed with GraphPad Prism v.8 software.

**Table 2:** Selected single nucleotide variants.

Gene	Locus	Function	Forward Primer	Reverse Primer	SNV	Position	Ref. ^a^	Frequency	MAF ^a^	Frequency
*AXIN1*	16p13.3	Cytoplasmic protein	ATGTCAGCCCCTT GTTTTTGCT	ATCTCGGGTAG CCGGTTTAGACT	rs370681	Intron	*C*	0.6295	*T*	0.3705
					rs12921862	Intron	*C*	0.887	*A*	0.113
					rs1805105	Exon 2	*A*	0.53508	*G*	0.4692
*TBX20*	7p14.2	Transcription factor	CTGTGCAGACT GTCGTCCTG	CACTGGCCTC TATTCCCCAC	rs4720169	Intron	*G*	0.3847	*A*	0.6153
*TBX1*	22q11.2	Transcription factor	AATGGGCGTCTT GTCTTCGC	GGGTCGCAGGG TCTGATTCC	rs41260844	Upstream	*C*	0.791	*T*	0.238
*MTHFR*	1p36	Folate metabolism	GGGCCTGAGCT GACAGAGAT	AACATGCTCCT CGGTGACAG	rs1801133	Exon 5	*G*	0.5473	*A*	0.4527
					rs1801131	Exon 8	*T*	0.808	*G*	0.192

Latin–American reference sequence (Ref.) ^a^ and minor allele frequency (MAF) ^a^ are shown.

**Table 3:** Comparison of the MS of the four genes analyzed between controls and cases.

Gene	MS Case Group	MS Control Group	*p*
*AXIN1*	73.81 (SD: ±35.60)	96.5 (SD: ±39.8)	0.036
*TBX20*	4.97 (SD: ±7.16)	11.17 (SD: ±21.8)	0.369
*TBX1*	4.1 (SD: ±5.67)	5.71 (SD: ±10.85)	0.523
*MTHFR*	4.48 (SD: ±4.63)	9.44 (SD: ±37.9)	0.041

MS—methylation status. Statistically significant difference: *p* < 0.05.

**Table 4:** Comparison of gene MS between ventricular septal defects and atrial septal defects.

Gene	MS VSD (*n* = 15)	MSD ASD (*n* = 7)	*p*
*AXIN1*	57.79 (SD: ±29.2)	108.14 (SD: ±20.74)	0.001
*TBX20*	2.23 (SD: ±2.17)	10.84 (SD: ±11.61)	0.017
*TBX1*	1.69 (SD: ±1.94)	9.56 (SD: ±7.47)	0.001
*MTHFR*	3.43 (SD: ±6.3)	6.72 (SD: ±6.3)	0.21

MS—methylation status. Statistically significant difference: *p* < 0.05.

**Table 5:** Comparison of MS between the four subgroups: case group with alternate allele, case group with wild-type allele, control group with alternate allele, and control group with wild-type allele.

		Case Group		Control Group		
Gene	SNV	MS Alternate Allele	MS Wild-Type	MS Alternate Allele	MS Wild-Type	*p*
*AXIN1*	rs370681	71.98 (SD: ±35.66)	75. 64 (SD: ±37.19)	98.19 (SD: ±49.09)	94.81 (SD: ±28.77)	0.651
	rs12921862	74.95 (SD: ±35.26)	66.58 (SD: ±21.52)	70.26 (SD: ±27.03)	101 (SD: ±41.64)	0.093
	rs1805105	74.09 (SD: ±34.78)	73.47 (SD: ±38.46)	99.015 (SD: ±44.78)	92.1 (SD: ±29.98)	0.574
*TBX20*	rs4720169	5.23 (SD: ±7.99)	2.37 (SD: ±0.43)	10.46 (SD: ±22.41)	12.10 (SD: ±21.71)	0.354
*TBX1*	rs41260844	5.97 (SD: ±4.1)	3.88 (SD: ±5.97)	4.84 (SD: ±7.2)	6.2 (SD: ±12.53)	0.064
*MTHFR*	rs1801133	4.85 (SD: ±6.63)	4.34 (SD: ±4.90)	37.34 (SD: ±85.9)	3.245 (SD: ±8.8)	0.004
	rs1801131	4.48 (SD: ±5.88)	4.46 (SD: ±2.68)	10.89 (SD: ±43.18)	4.52 (SD: ±4.76)	0.007

MS—methylation status. Statistically significant difference: *p* < 0.0471 by Bonferroni inequalities.

## Data Availability

Not applicable.
